# 高通量固相萃取-超高效液相色谱-高分辨质谱法测定血清中29种全氟/多氟化合物和22种有机磷酸酯及其二酯代谢物

**DOI:** 10.3724/SP.J.1123.2025.07008

**Published:** 2026-07-08

**Authors:** Shiqi ZHAO, Hao DING, Xuwenqi ZHANG, Minmin HOU, Tingting ZHOU, Yali SHI, Yaqi CAI

**Affiliations:** 1. 国科大杭州高等研究院环境学院，全省新污染物环境与健康重点实验室，浙江 杭州 310024; 1. Zhejiang Key Laboratory of Environment & Health of New Pollutants，School of Environment，Hangzhou Institute for Advanced Study，University of Chinese Academy of Sciences，Hangzhou 310024，China; 2. 中国科学院生态环境研究中心，环境化学与生态毒理学国家重点实验室，北京 100085; 2. State Key Laboratory of Environment Chemistry and Ecotoxicology，Research Center for Eco-Environmental Sciences，Chinese Academy of Sciences，Beijing 100085，China; 3. 中国科学院大学，北京 100049; 3. University of Chinese Academy of Sciences，Beijing 100049，China; 4. 浙江省生态环境科学设计研究院，全省新污染物环境与健康重点实验室，浙江 杭州 310007; 4. Zhejiang Key Laboratory of Environment and Health of New Pollutants，Ecological and Environmental Science and Research Institute of Zhejiang Province，Hangzhou 310007，China

**Keywords:** 全氟/多氟化合物, 有机磷酸酯, 96孔板, 超高效液相色谱-高分辨质谱, 血清, per- and polyfluoroalkyl substances （PFAS）, organophosphate esters （OPEs）, 96-wellplate, ultra performance liquid chromatography-high resolution mass spectrometry （UPLC-HRMS）, serum

## Abstract

监测血液中的污染物是评估其人体暴露水平和健康风险的重要依据。全氟/多氟化合物（PFAS）、有机磷酸酯（OPEs）及其二酯代谢物（di-OPEs）作为广泛共存于环境并具有显著毒性效应的污染物，对其在人体内暴露水平的监测具有重要意义。本研究基于96孔板固相萃取柱前处理方法，通过比较3种萃取柱，并对前处理步骤进行优化，建立了检测血清样品中29种PFAS、17种OPEs和5种di-OPEs的超高效液相色谱-高分辨质谱法（UPLC-HRMS）。使用Phree PLR 96孔板萃取净化，向其中依次加入300 µL含1%甲酸的乙腈，100 µL血清样品，最后用100 µL含1%甲酸的乙腈溶液洗脱。洗脱液真空浓缩后使用UPLC-HRMS测定目标化合物。PFAS和di-OPEs选用负离子模式，OPEs选用正离子模式，在全扫描/数据依赖的二级扫描模式（Full MS/ddMS^2^）下测定，使用内标法进行校正。结果表明，在优化条件下，目标物在0.05~50 ng/mL内具有良好的线性关系（*R* ^2^>0.991），方法检出限（MDL）为0.000 120~0.274 ng/mL。此方法具有操作简单、萃取时间短、高通量等优点。应用该方法对2024年采集的济南地区15份血清样品进行测定，结果表明29种PFAS总量为6.71~379 ng/mL，其中PFOA中位含量为8.96 ng/mL，PFOS中位含量为4.07 ng/mL。17种OPEs总含量为0.015 0~10.5 ng/mL，中位含量为2.81 ng/mL。5种di-OPEs的总含量为<MDL~0.443 ng/mL，中位含量为0.015 0 ng/mL，说明这些污染物在人体血液中的复合暴露状况及其引起的潜在健康风险应该引起重视。

## Abstract (en)

Monitoring pollutants in human blood is a crucial basis for assessing human exposure levels and health risks. Per- and polyfluoroalkyl substances （PFAS）， organophosphate esters （OPEs）， and their diester metabolites （di-OPEs） are widespread environmental co-contaminants with significant toxic effects， making it crucial to monitor their internal human exposure levels. However， existing studies have predominantly investigated these substances in isolation， lacking comprehensive research that simultaneously quantifies PFAS， OPEs， and di-OPEs in human serum. Based on the pretreatment method of 96-well solid phase extraction columns， this study compared three extraction columns and optimized the pretreatment steps to establish an ultra performance liquid chromatography-high resolution mass spectrometry （UPLC-HRMS） method for 29 PFAS， 17 OPEs， and 5 di-OPEs in human serum samples. Phree PLR 96-Wellplate was used as a cleanup plate. 300 µL of 1% formic acid in acetonitrile， 100 µL of the serum sample， and internal standards were added to the cleanup device in sequence. After standing for 5 minutes， the 96-well plate positive pressure device was used to press the mixture into the 96-well collection plate. Finally， the sample was eluted with 100 µL of 1% formic acid in acetonitrile. The eluate was collected and concentrated for the detection of PFAS， OPEs， and di-OPEs by UPLC-HRMS. PFAS and OPEs were detected using an Acclaim RSLC 120 C18 column， while di-OPEs were detected using an Acquity UPLC BEH C18 column. Both were subjected to gradient elution with methanol and 5 mmol/L ammonium acetate in water as the mobile phases. Sample ionization was performed using a heated electrospray ionization source （H-ESI）. PFAS and di-OPEs were analyzed in negative ion mode， while OPEs were analyzed in positive ion mode. Data acquisition was conducted in full-scan/data-dependent tandem mass spectrometry （Full MS/ddMS^2^） mode. Quantification was achieved using the internal standard calibration method to ensure measurement accuracy. The results showed that under the optimized conditions， the target compounds had good linear relationships in the range of 0.05–50 ng/mL （*R* ^2 ^> 0.99）， and the method detection limits （MDLs） of 29 PFAS， 17 OPEs and 5 di-OPEs were 0.000 120–0.274 ng/mL， 0.011 0–0.250 ng/mL， and 0.012 0–0.220 ng/mL， respectively， and the spiked recoveries were between 45.9% and 147.8%. The relative standard deviations （RSDs） were 1.2%–29.0%. Most PFAS and di-OPEs had matrix enhancement effects， and most OPEs had matrix inhibition effects. Among them， hexafluoropropylene oxide dimer acid （GenX） （196.5%） and trimethylphenyl phosphate （TMPP） （54.6%） had significant matrix enhancement and inhibition effects， respectively， which could be corrected with appropriate internal standards. The recoveries of these two substances after correction were 127.3% and 78.7%， respectively， which met the analysis requirements. The proposed approach offers significant practical benefits， combining straightforward operation with shortened extraction time and enhanced throughput， which was validated through analysis of 15 human serum samples collected in Jinan in 2024. The total contents of 29 PFAS were 6.71–379 ng/mL， with a median value of 22.9 ng/mL. Eight PFAS were detected with a detection frequency of 100.0%， with median contents of perfluorooctanoic acid （PFOA） and perfluorooctane sulfonate （PFOS） reaching 8.96 ng/mL and 4.07 ng/mL， respectively. The total contents of 17 OPEs were 0.015 0–10.5 ng/mL， with a median value of 2.81 ng/mL. The most frequently detected OPEs， with detection frequencies exceeding 60.0%， were triethyl phosphate （TEP）， tri-*n*-butyl phosphate （TnBP）， and triphenyl phosphate （TPHP）. The total contents of 5 di-OPEs were <MDL–0.443 ng/mL， with a median value of 0.015 0 ng/mL. Therefore， the combined exposure to these pollutants in human blood and its potential health risks demand serious attention.

全氟/多氟化合物（per- and polyfluoroalkyl substances， PFAS）是一类人工合成的有机氟化合物^［1］^，因其独特的疏水疏油性、化学稳定性、热稳定性和表面活性而被广泛应用于多类消费品中，像常见的不粘锅具、化妆品，此外PFAS还被用于消防泡沫、人造草坪等众多产品中^［2］^。PFAS具有强稳定性和难降解性，目前已在水^［3］^、土^［4］^、气^［5］^、人体样本^［6］^等中被广泛检出。2009年全氟辛烷磺酸（PFOS）被正式列入《斯德哥尔摩公约》的持久性有机污染物名单中，全氟辛酸（PFOA）和全氟己烷磺酸（PFHxS）也分别在2019年和2022年被列入斯德哥尔摩公约的附件A（消除类）名单中^［7，8］^。已有研究表明，人体多种健康问题与PFAS暴露和累积有关^［9］^，包括发育毒性^［10］^、内分泌干扰^［11］^、肝毒性^［12，13］^、神经毒性^［14］^、肾毒性^［15］^和免疫毒性^［16］^。

有机磷酸酯（organophosphate esters， OPEs）是一类新污染物，在多溴二苯醚（PBDEs）和六溴环十二烷（HBCD）等传统溴代阻燃剂被列入斯德哥尔摩公约后，OPEs作为阻燃剂和增塑剂，广泛应用于建筑材料、电子产品和纺织品等各类消费品中^［17，18］^，其消费量也随之逐年增长。作为一类添加剂，OPEs很容易通过磨损等方式释放到环境中，目前已经在多种环境介质中检出^［17］^，导致OPEs在人类常见的暴露来源中普遍存在。部分OPEs进入人体后易被代谢成有机磷酸二酯（organophosphate diesters， di-OPEs），并经尿液排出。此外，某些di-OPEs本身具有工业应用或者会通过OPEs在自然环境中降解产生^［19，20］^。目前，已经在灰尘^［21］^、食物^［22］^和饮用水^［23］^中检出di-OPEs，提示人体存在直接暴露于di-OPEs的风险。OPEs被证实具有发育毒性^［24］^、内分泌干扰效应^［25，26］^和致癌性^［27］^等。

PFAS、OPEs和di-OPEs会通过皮肤接触、呼吸、灰尘摄入、饮食和饮水等途径进入人体，威胁到人类健康。Li等^［28］^对2022年河北省石家庄市普通人群的血液进行检测发现8种OPEs检出率超过60%，6种PFAS被广泛检出，其中PFHxS和全氟壬酸（PFNA）检出率达到100%。对山东省济南市中老年人血液和尿液中多种环境污染物的检测结果发现，血液中PFOA含量最高（中位数：7.68 µg/L），其次是PFOS（5.24 µg/L）和磷酸三丁酯（TBP）（1.03 µg/L）。尿液中磷酸二（2-氯乙基）酯（BCEP）具有较高的含量（中位数：937.5 µg/g，尿肌酐校正浓度）^［29］^。相比于尿液样本，血液样本的获取虽然相对复杂，但是作为人体内流动在各个器官和组织的循环液，对其中污染物质的研究有助于了解其在人体内的分布和富集^［30］^。血液中污染物水平可以反映出一个人在过去一段时间内通过各种途径接触到污染物的总量，以及同时接触到多种化合物对人体产生的整体影响，因此，是一种可以全面衡量人体受到多类物质影响程度的重要指标^［31］^。特别是针对PFAS，其在生物体内主要累积在血清中^［5］^；大部分OPEs倾向于与血浆蛋白结合，如血清白蛋白^［32］^。故使用血清作为检测样本更具有科学意义。因此，建立一种使用微量血清能实现多类污染物（如PFAS、OPEs和di-OPEs）同时快速萃取方法显得尤为重要。

对于血清样本中PFAS、OPEs和di-OPEs的前处理方法通常包含提取和净化两个步骤，常见的提取方法有液液萃取^［33-36］^、沸石-索氏萃取法^［37］^等。常见的净化方法有固相萃取^［38］^和固相微萃取^［39］^。肖锴婷等^［38］^使用乙腈为萃取剂，通过超声萃取3次，每次30 min，最后用C18作为吸附剂净化萃取液的方法检测人体血清中的PFAS。侯敏敏等^［40］^使用乙腈作为萃取液，重复萃取3次，萃取时长超过12 h，萃取所得上清液使用ENVI-18小柱净化。这些提取方法都存在着萃取时间长、次数多且需要样品量多的缺陷，且目前尚未有血液中PFAS和OPEs同时提取净化的方法报道。由于血清样本是珍贵的生物样本之一，建立一种能够使用微量血清样本、前处理时间短且能满足检测PFAS、OPEs和di-OPEs 3种化合物的方法十分必要。因此，本研究基于96孔板，建立了高通量萃取人体微量血清中PFAS、OPEs和di-OPEs的方法，为其环境与健康风险评估提供了方法基础。

## 1 实验部分

### 1.1 仪器、试剂与材料

Ultimate 3000 HPLC高效液相色谱仪、Orbitrap Exploris^TM ^240质谱检测系统（美国Thermo Fisher公司）；Cleanert M96生物样品前处理仪SPE-M96（中国Agela飞诺美公司）；冷冻离心浓缩仪Auto R1-Plus（中国吉艾姆公司）；Phree PLR 96孔板（30 mg）和Cleanert PWCX 96孔板（30 mg）均购自中国Agela飞诺美公司；Anavo HMR 96孔板（20 mg）购自北京纳鸥科技有限公司。

甲醇和乙腈（色谱纯，美国Thermo Fisher公司）；甲酸（99%，美国Fluka公司）；蒸馏水（4.5 L，北京屈臣氏蒸馏水有限公司）；乙酸铵（97%，美国Alfa Aesar公司）。

29种PFAS标准品及同位素内标购自加拿大Wellington Laboratories。OPEs标准品及内标分别购自德国Dr. Ehrenstorfer公司、加拿大Toronto Research Chemicals公司和加拿大Wellington Laboratories。5种di-OPEs标准品及5种同位素内标全部购自加拿大Toronto Research Chemicals公司。化合物详细情况见表1。

**表 1:** 29种PFAS、17种OPEs及5种di-OPEs的信息

Compound	Abbreviation	Chinese name	Mass labeled standard
Perfluorobutanoic acid	PFBA	全氟丁酸	^13^C_4_-PFBA
Perfluoropentanoic acid	PFPeA	全氟戊酸	^13^C_4_-PFBA
Perfluorohexanoic acid	PFHxA	全氟己酸	^13^C_2_-PFHxA
Perfluoroheptanoic acid	PFHpA	全氟庚酸	^13^C_4_-PFOA
Perfluorooctanoic acid	PFOA	全氟辛酸	^13^C_4_-PFOA
Perfluorononanoic acid	PFNA	全氟壬酸	^13^C_5_-PFNA
Perfluorodecanoic acid	PFDA	全氟癸酸	^13^C_2_-PFDA
Perfluoroundecanoic acid	PFUnDA	全氟十一烷酸	^13^C_2_-PFUnDA
Perfluorododecanoic acid	PFDoDA	全氟十二烷酸	^13^C_2_-PFDoDA
Perfluorotridecanoic acid	PFTrDA	全氟十三烷酸	^13^C_2_-PFDoDA
Perfluorotetradecanoic acid	PFTeDA	全氟十四烷酸	^13^C_2_-PFDoDA
Perfluorobutane sulfonate	PFBS	全氟丁烷磺酸	^18^O_2_-PFHxS
Perfluoropentane sulfonate	PFPeS	全氟戊烷磺酸	^18^O_2_-PFHxS
Perfluorohexane sulfonate	PFHxS	全氟己烷磺酸	^18^O_2_-PFHxS
Perfluoroheptane sulfonate	PFHpS	全氟庚烷磺酸	^18^O_2_-PFHxS
Perfluorooctane sulfonate	PFOS	全氟辛烷磺酸	^13^C_4_-PFOS
Perfluorononane sulfonate	PFNS	全氟壬烷磺酸	^13^C_4_-PFOS
Perfluorodecane sulfonate	PFDS	全氟癸烷磺酸	^13^C_4_-PFOS
6∶2 Chlorinated polyfluoroalkyl ether sulfonic acid	6∶2 Cl-PFESA	6∶2氯化多氟醚磺酸	^13^C_4_-PFOS
8∶2 Chlorinated polyfluoroalkyl ether sulfonic acid	8∶2 Cl-PFESA	8∶2氯化多氟醚磺酸	^13^C_4_-PFOS
10∶2 Chlorinated polyfluoroalkyl ether sulfonic acid	10∶2 Cl-PFESA	10∶2氯化多氟醚磺酸	^13^C_4_-PFOS
6∶2 Fluorotelomer sulfonate	6∶2 FTS	6∶2氟调聚物磺酸	^18^O_2_-PFHxS
Perfluorobutylsulphonamide	FBSA	全氟丁基磺酰胺	^18^O_2_-PFHxS
Perfluorohexanesulfonamide	FHxSA	全氟己烷磺酰胺	^18^O_2_-PFHxS
Perfluorooctanesulfonamide	FOSA	全氟辛基磺酰胺	^13^C_4_-PFOS
*N*-Ethylperfluoro-1-octanesulfonamidoacetic acid	*N*-EtFOSAA	*N*-乙基全氟-1-辛烷磺酰胺基乙酸	^13^C_2_-PFDA
*N*-Methylperfluoro-1-octanesulfonamidoacetic acid	*N*-MeFOSAA	*N*-甲基全氟-1-辛烷磺酰胺基乙酸	^13^C_2_-PFDA
Hexafluoropropylene oxide dimer acid	GenX	六氟环氧丙烷二聚体	^13^C_4_-PFOA
Sodium dodecafluoro-3*H*-4，8-dioxanonanoate	ADONA	十二氟-3*H*-4，8-二氧壬酸钠	^13^C_4_-PFOA
Trimethyl phosphate	TMP	磷酸三甲酯	TMP-d_9_
Triethyl phosphate	TEP	磷酸三乙酯	TEP-d_15_
Tripropyl phosphate	TPrP	磷酸三丙酯	TPrP-d_21_
Tri-*n*-butyl phosphate	TnBP	磷酸三正丁酯	TnBP-d_27_
Tris（2-ethylhexyl） phosphate	TEHP	磷酸三（2-乙基己基）酯	TEHP-d_51_
Tri（2-chloroethyl） phosphate	TCEP	磷酸三（2-氯乙基）酯	TCEP-d_12_
Tri（1-chloro-2-propyl） phosphate	TCIPP	磷酸三（1-氯-2-丙基）酯	TCIPP-d_18_
Tri（1，3-dichloro-2-propyl） phosphate	TDCPP	磷酸三（1，3-二氯-2-丙基）酯	TDCPP-d_15_
2，2-Bis（chloromethyl） trimethylene bis（bis（2-chloroethyl） phosphate）	V6	2，2-双（氯甲基）三亚甲基双 （双（2-氯乙基）磷酸酯）	TCIPP-d_18_
Tri（2-butoxyethyl） phosphate	TBOEP	磷酸三丁氧乙酯	TBOEP-d_27_
Tri-phenyl phosphate	TPHP	磷酸三苯酯	TPHP-d_15_
Trimethylphenyl phosphate	TMPP	磷酸三甲苯酯	TPHP-d_15_
3-Isopropylphenyl diphenyl phosphate	3IPPDPP	3-异丙基苯基二苯基磷酸酯	TPHP-d_15_
Cresyl diphenyl phosphate	CDPP	磷酸甲苯二苯酯	TPHP-d_15_
Bis（3-*tert*-butylphenyl） phenyl phosphate	B3tBPPP	磷酸双（3-叔丁基苯基）苯酯	TPHP-d_15_
Bis（3-isopropylphenyl） phenyl phosphate	B3IPPPP	磷酸双（3-异丙基苯基）苯酯	TPHP-d_15_
3-*tert*-Butylphenyl diphenyl phosphate	3tBPDPP	3-叔丁基苯基二苯基磷酸酯	TPHP-d_15_
Dibutyl phosphate	DBP	磷酸二丁酯	DnBP-d_18_
Bis（2-ethylhexyl） phosphate	BEHP	磷酸氢双（2-乙基己基）酯	BEHP-d_34_
Di-phenyl phosphate	DPHP	磷酸氢二苯酯	DPHP-d_10_
Di-*o*-tolyl-phosphate	BMPP	磷酸二邻甲苯基酯	BMPP-d_14_
Bis（2-butoxyethy） phosphate	BBOEP	双（丁氧基乙基）磷酸酯	BBOEP-d_8_

29种PFAS混合标准储备液（100 ng/mL）采用甲醇配制，置于-20 ℃的冰箱中保存。17种OPEs的混合标准储备液（1 000 ng/mL）采用乙腈配制，置于-20 ℃的冰箱中保存。5种di-OPEs的混合标准储备液（1 000 ng/mL）采用甲醇配制，置于-20 ℃的冰箱中保存。

血清样本：2024年采集自山东济南地区的健康志愿者，已通过伦理委员会审查并批准，编号：HeB1RBS2023-002。

### 1.2 样品前处理

将血清样品从冰箱里取出后放置在常温下，待自然解冻，取Phree PLR 96孔板（30 mg），向其中依次加入300 µL 1%甲酸乙腈、100 µL血清样本、10 µL内标混合溶液（PFAS 20 ng/mL、OPEs 100 ng/mL、di-OPEs 100 ng/mL），静置老化5 min以充分混合，将96孔板放在96孔板正压装置下，加压，收集洗脱液，再向小柱内加入100 µL 1%甲酸乙腈洗脱，加压，将两次洗脱液合并在96孔收集板内。使用冷冻真空浓缩仪将洗脱液浓缩至近干后，用甲醇定容至100 µL，涡旋后转移至放有250 µL玻璃内插管的进样小瓶中待测。

### 1.3 仪器检测条件

#### 1.3.1 色谱条件

对PFAS的检测使用Acclaim RSLC 120 C18液相色谱柱（100 mm×2.1 mm，2.2 µm，美国Thermo Fisher公司）进行样品分离，柱温35 ℃，流动相A为甲醇，流动相B为5 mmol/L乙酸铵缓冲溶液；流速为0.35 mL/min；梯度洗脱程序：0~1 min，95%B；1~3 min，95%B~30%B；3~9 min，30%B~1%B；9~10 min，1%B；10~10.1 min，1%B~95%B；10.1~13 min，95%B。

对OPEs的检测使用的色谱柱、流动相、柱温和流速参数与PFAS相同。梯度洗脱程序：0~1 min，90%B~60%B；1~3 min，60%B~10%B；3~3.1 min，10%B~0B；3.1~8.1 min，0B；8.1~8.6 min，0B~90%B；8.6~14 min，90%B。

对di-OPEs的检测使用Acquity UPLC BEH C18液相色谱柱（100 mm×2.1 mm，1.7 µm，美国Waters公司）进行样品分离，柱温35 ℃，流动相A为甲醇，流动相B为5 mmol/L乙酸铵缓冲溶液；流速为0.35 mL/min，梯度洗脱程序：0~1 min，90%B~60%B；1~3 min，60%B~10%B；3~3.1 min，10%B~0B；3.1~8.1 min，0B；8.1~8.6 min，0B~90% B；8.6~14 min，90%B。

#### 1.3.2 质谱条件

离子源为加热电喷雾离子源（H-ESI），离子传输管温度为 320 ℃，雾化温度350 ℃；PFAS和di-OPEs设置喷雾电压为3.4 kV（负离子模式）；OPEs设置喷雾电压为2.3 kV（正离子模式）；鞘气流速为45 Arb；辅助气流速为10 Arb。数据采集模式采用全扫描/数据依赖的二级扫描模式（full mass-data dependent scans，Full MS/ddMS^2^）方式，根据目标化合物的*m/z*范围，将扫描范围设置为*m/z* 70.0~1 050.0，RF Lens为80%，一级质谱分辨率为120 000，自动增益控制目标离子数（automatic gain control target，AGC target）为1×10^6^；二级质谱分辨率为15 000，AGC target为1×10^5^；高能碰撞解离 (HCD)碰撞能为20%、40%、60%。

### 1.4 质量保证与质量控制

在整个采样和实验过程中尽量避免塑料制品，对无法避免的塑料制品使用乙腈和甲醇分别超声15 min进行清洗。样品前处理时每10个样品中加2个程序空白样品以监测前处理过程中可能引入的污染。使用内标法对目标物质进行定量，仪器检测时，每12个样品加一针标准溶液（2 ng/mL）作为质控样，以检测仪器信号稳定，若质控样测定结果与真实值的偏差大于±20%，则须重新绘制标准曲线。使用Anavo Ghost peak-Trapper（50 mm×2.1 mm，北京纳鸥科技有限公司）鬼峰捕集柱去除仪器污染，每12个样品加一针甲醇溶液以监测仪器空白。

## 2 结果与讨论

### 2.1 固相萃取柱的选择

96孔除磷脂板的主要作用是去除血液中血浆蛋白和磷脂，具有快速且高通量的特性，不同填料以及填料含量对蛋白和磷脂的去除效果有所不同。本研究分别比较了Cleanert PWCX 96孔板（30 mg）、Phree PLR 96孔板（30 mg）和Anavo HMR 96孔板（20 mg）3种96孔板对血清中PFAS、OPEs和di-OPEs的加标回收率，结果见图1。使用Cleanert PWCX 96孔板、Phree PLR 96孔板和Anavo HMR 96孔板时，回收率在60.0%~130.0%的目标化合物数量分别为39种、42种和31种，优先选用目标化合物回收率达到可接受范围数量较多的96孔板。使用Cleanert PWCX 96孔板时，7种目标物的回收率低于60.0%，15种目标物的相对标准偏差（RSD）在25.0%以上；而在使用Phree PLR 96孔板时仅有4种目标物的回收率低于60.0%（TMPP、3IPPDPP、CDPP、3tBPDPP），2种目标物的RSD在25.0%以上（TnBP和DPHP）。因此我们最终选择Phree PLR 96孔板（30 mg）进行血清样本的前处理。

**图1:**
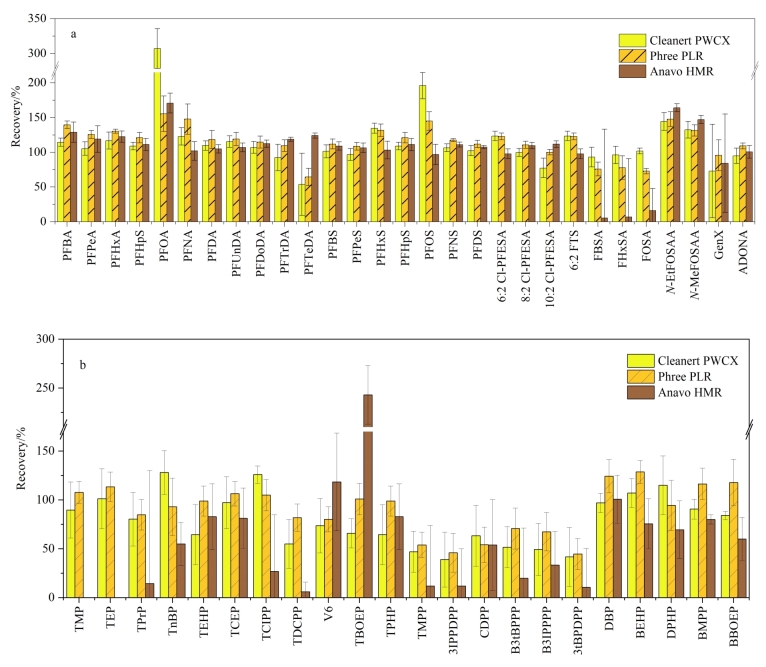
采用3种96孔板时（a）PFAS、（b）OPEs和di-OPEs的回收率（*n*=4）

### 2.2 前处理流程的优化

本研究对比了两种前处理方法对目标物的回收率：方法一先加入1%甲酸乙腈再加入血清与内标；方法二先加入血清与内标再加入1%甲酸乙腈。后续步骤两种方法相同，充分混合后静置5 min，正压收集洗脱液，再用1%甲酸乙腈冲洗萃取柱，收集两次洗脱液。加标回收率结果见图2。使用方法一目标物的加标回收率为44.7%~155.5%，RSD为2.2%~29.2%；使用方法二目标物的加标回收率为20.4%~147.2%，RSD为3.5%~49.6%。整体上，方法二的稳定性不如方法一，例如对于PFOA（方法一RSD为25.4%，方法二RSD为49.6%）和PFOS（方法一RSD为13.2%，方法二RSD为24.4%）这两种在血清中广泛存在的物质尤为明显。且在实验过程中，使用方法二在复溶后，溶液中会有明显沉淀，可能是血液中磷脂去除不完全所致。因此，我们最终选择方法一进行样本前处理。

**图2:**
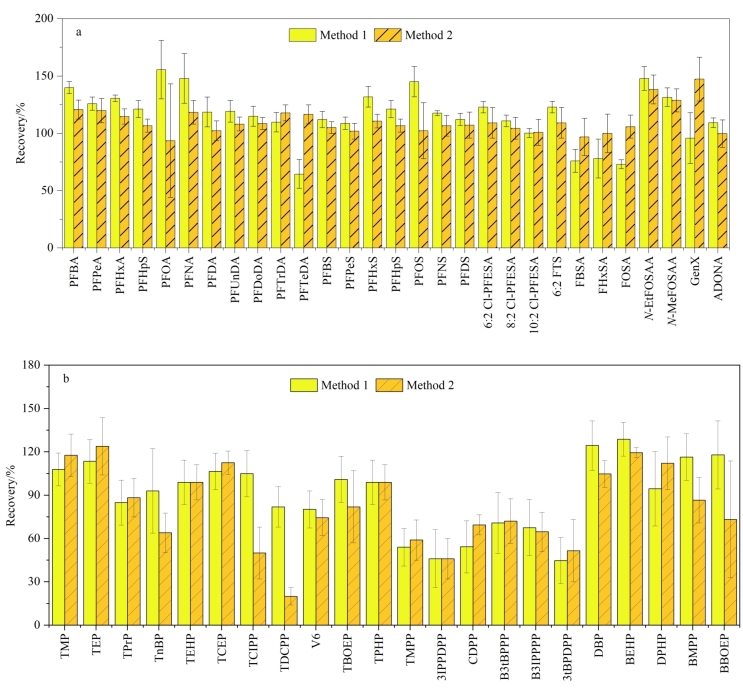
采用2种不同前处理方法时（a）PFAS、（b）OPEs和di-OPEs的回收率（*n*=4）

### 2.3 基质效应

为评估前处理后残存杂质对目标物定量的影响，本研究取不添加任何目标化合物和内标的血清样品，根据优化好的前处理方法进行萃取净化，将最后获得的萃取液混合得到空白基质溶液，取基质空白加入目标化合物及其内标来评估Phree PLR 96孔板（30 mg）血清前处理方法的基质效应（matrix effect，ME），目标化合物的基质效应计算方法见公式（1）：


$$ME=\frac{A_{1}-A_{0}}{A_{2}}\times 100\%$$ （1）

其中，*A*_0_为基质空白中目标化合物的响应峰面积；*A*_1_为加标基质中目标物的响应峰面积；*A*_2_为纯溶剂甲醇中目标物的响应峰面积。

ME<100%，表示有基质抑制效应；ME>100%，表示有基质增强效应；ME=100%，表示无基质效应。血清中这51种目标物质的基质效应为54.6%~196.5%，大部分PFAS和di-OPEs存在基质增强效应，大部分OPEs存在基质抑制效应。其中GenX（196.5%）和TMPP（54.6%）分别存在显著的基质增强和抑制效应，不过可以用合适的稳定性同位素内标（^13^C_4_-PFOA（111.2%）和TPHP-d_15_（70.3%））进行校正，校正后目标物质的回收率分别为127.3%和78.7%，整体可以满足分析的要求。

### 2.4 线性范围与检出限

以甲醇为溶剂，配制与样品浓度相对应的质量浓度（0.05、0.1、0.2、0.5、1、2、5、10、20、50 ng/mL）的PFAS、OPEs和di-OPEs混合标准溶液，每次测样品前用相同的仪器条件按顺序进行检测，以目标化合物与对应内标物峰面积之比为纵坐标（*y*），以目标物质量浓度为横坐标（*x*，ng/mL）绘制标准曲线。结果表明，所有目标化合物均在该范围内表现出良好的线性关系（相关系数*R* ^2^>0.991）。对流程空白中可以检出的目标物，将测定结果换算成浓度，计算7次结果的标准偏差，以3倍标准偏差作为方法检出限（MDL）。对实验过程空白中未检出的物质，以低水平加标（0.5 ng/mL），按照样品前处理方法进行处理，平行测定7次，计算3倍标准偏差作为MDL。最终所得的PFAS、OPEs和di-OPEs的MDL分别为0.000 120~0.274 ng/mL、0.011 0~0.250 ng/mL和0.012 0~0.220 ng/mL，具体结果见表2。

**表 2:** PFAS、OPEs及di-OPEs的线性方程、线性范围、相关系数和方法检出限

Compound	Linear equation	Linear range/（ng/mL）	*R* ^2^	MDL/（ng/mL）
PFBA	*y*=0.528*x*-0.0123	0.1-50	0.991	0.0802
PFPeA	*y*=0.707*x*-0.000841	0.1-50	0.991	0.0540
PFHxA	*y*=0.582*x*-0.00150	0.05-50	0.996	0.0130
PFHpA	*y*=0.418*x*+0.000301	0.05-50	0.993	0.00990
PFOA	*y*=0.575*x*-0.00126	0.05-50	0.999	0.000120
PFNA	*y*=0.0556*x*-0.000519	0.2-50	0.998	0.130
PFDA	*y*=0.579*x*-0.00345	0.1-50	0.997	0.077
PFUnDA	*y*=0.562*x*+0.00175	0.2-50	0.995	0.160
PFDoDA	*y*=0.570*x*-0.00146	0.05-50	0.998	0.0290
PFTrDA	*y*=0.514*x*+0.000577	0.05-50	0.997	0.0450
PFTeDA	*y*=0.424*x*-0.00457	0.05-50	0.994	0.0190
PFBS	*y*=0.644*x*-0.00139	0.05-50	0.998	0.00500
PFPeS	*y*=0.650*x*+0.000313	0.05-50	0.998	0.00240
PFHxS	*y*=0.662*x*+0.00259	0.05-50	0.998	0.0130
PFHpS	*y*=0.648*x*+0.00172	0.05-50	0.999	0.00740
PFOS	*y*=0.578*x*-0.00222	0.05-50	0.999	0.00270
PFNS	*y*=0.542*x*-0.00373	0.05-50	0.998	0.00460
PFDS	*y*=0.510*x*-0.00198	0.2-50	0.998	0.140
6∶2 Cl-PFESA	*y*=0.432*x*-0.000948	0.1-50	0.995	0.0670
8∶2 Cl-PFESA	*y*=0.360*x*-0.00246	0.05-50	0.998	0.00570
10∶2 Cl-PFESA	*y*=0.296*x*-0.00150	0.2-50	0.995	0.190
6∶2 FTS	*y*=0.0782*x*+0.000669	0.05-50	0.993	0.00390
FBSA	*y*=0.600*x*-0.00198	0.05-50	0.999	0.0110
FHxSA	*y*=0.602*x*-0.000426	0.05-50	0.998	0.0060
FOSA	*y*=0.495*x*-0.000975	0.05-50	0.996	0.0120
*N*-EtFOSAA	*y*=0.170*x*-0.00548	0.2-50	0.995	0.130
*N*-MeFOSAA	*y*=0.174*x*-0.00444	0.05-50	0.996	0.0180
GenX	*y*=0.0153*x*-0.00109	0.1-50	0.991	0.274
ADONA	*y*=0.704*x*-0.00190	0.05-50	0.996	0.00580
TMP	*y*=0.585*x*-0.935	0.2-20	0.995	0.190
TEP	*y*=0.0770*x*-0.00810	0.2-20	0.991	0.130
TPrP	*y*=0.0815*x*-0.00465	0.5-20	0.992	0.250
TnBP	*y*=0.0974*x*-0.00133	0.05-20	0.999	0.0310
TEHP	*y*=0.112*x*-0.00134	0.05-20	0.999	0.0260
TCEP	*y*=0.122*x*-0.000211	0.05-20	0.999	0.0440
TCIPP	*y*=0.105*x*-0.0133	0.5-20	0.999	0.240
TDCPP	*y*=0.0771*x*-0.0105	0.2-20	0.998	0.200
V6	*y*=0.0366*x*-0.00183	0.05-20	0.998	0.0170
TBOEP	*y*=0.159*x*-0.00203	0.05-20	0.996	0.0110
TPHP	*y*=0.189*x*-0.000595	0.05-20	0.999	0.0110
TMPP	*y*=0.187*x*-0.00116	0.05-20	0.999	0.0170
3IPPDPP	*y*=0.139*x*-0.000926	0.05-20	0.999	0.0150
CDPP	*y*=0.0495*x*-0.00171	0.05-20	0.998	0.0370
B3tBPPP	*y*=0.131*x*-0.00171	0.05-20	0.999	0.0190
B3IPPPP	*y*=0.150*x*-0.00254	0.05-20	0.998	0.0170
3tBPDPP	*y*=0.135*x*-0.000247	0.05-20	0.999	0.0320
DBP	*y*=0.207*x*-0.00663	0.2-20	0.995	0.140
BEHP	*y*=0.112*x*-0.000163	0.5-20	0.993	0.220
DPHP	*y*=0.123*x*-0.00508	0.5-20	0.992	0.210
BMPP	*y*=0.104*x*-0.00208	0.05-20	0.996	0.0270
BBOEP	*y*=0.069*x*-0.00273	0.05-20	0.993	0.0120

*y*： peak area ratio of the target compound to its corresponding internal standard； *x*： mass concentration， ng/mL.

### 2.5 准确度与精密度

通过对实际血清混合样品进行加标回收试验考察方法的准确度和精密度。设置三组不同浓度加标试验，第一组添加0.2 ng/mL PFAS、0.5 ng/mL OPEs和0.5 ng/mL di-OPEs的混合标准溶液，第二组添加2 ng/mL PFAS、5 ng/mL OPEs和5 ng/mL di-OPEs的混合标准溶液，第三组添加4 ng/mL PFAS、10 ng/mL OPEs和10 ng/mL di-OPEs的混合标准溶液，每个水平设置4个平行样品。

结果见表3，第一组加标回收率为45.9%~126.8%，RSD为1.2%~28.5%，第二组加标回收率为48.0%~147.8%，RSD为2.2%~22.1%；第三组加标回收率为60.3%~143.3%，RSD为1.3%~29.0%；对于GenX，由于MDL较高（0.274 ng/mL），因此在0.2 ng/mL的添加水平下无法评估GenX的萃取回收率。PFBA、PFOA、PFNA、PFHxS和PFOS在前两组加标水平下呈现回收率偏高的现象（139.7%~279.8%）。我们分析认为，这主要源于其自身较高的血清本底浓度（较难获取不含上述PFAS的空白血清）。在此条件下，较小或持平的加标增量使得对本底值与加标后值的微小测量误差在计算过程中被显著放大，从而推高了回收率计算结果。当PFAS加标水平提升至4 ng/mL时（第三组），除本底浓度最高的PFOA（166.1%）外，其余物质的回收率均恢复至可接受范围（60.3%~119.7%），且RSD为1.4%~26.5%。尽管PFOA的回收率计算值仍偏高，但其对应内标的绝对回收率正常（82.7%），表明分析流程本身稳定可靠。第三组TEHP回收率略偏高（159.3%），源于其氘代内标（TEHP-d_15_）的绝对回收率低于TEHP本身的绝对回收率，这可能与氘代标记引入的微小物理化学性质差异有关。以上结果证明，该方法在实际应用浓度范围内具有良好的准确度与精密度，能够满足检测要求。

**表 3:** PFAS、OPEs及di-OPEs在人体血清中3个水平下的加标回收率（*n*=4）

Compound	Low		Medium		High	
	Recovery/%	RSD/%	Recovery/%	RSD/%	Recovery/%	RSD/%
PFBA	105.6	16.4	139.7	5.2	85.3	2.4
PFPeA	120.9	5.5	125.7	5.8	87.4	2.6
PFHxA	104.1	3.6	130.5	2.9	85.1	4.0
PFHpA	99.0	11.1	121.2	7.5	75.3	3.6
PFOA	279.8	14.6	156.4	25.2	166.1	18.8
PFNA	162.2	16.8	147.8	21.7	90.2	1.9
PFDA	118.6	15.3	118.5	13.0	90.7	6.5
PFUnDA	125.2	12.5	119.2	9.4	83.3	2.4
PFDoDA	106.3	7.4	114.8	8.7	77.6	5.1
PFTrDA	121.4	12	109.7	8.4	89.3	2.8
PFTeDA	90.7	13.1	64.4	12.7	70.3	5.0
PFBS	96.2	3.6	112.0	7.1	84.4	1.4
PFPeS	104.9	5.5	108.7	5.4	83.3	2.9
PFHxS	163.8	3.5	131.8	9.0	93.4	4.8
PFHpS	104.0	4.9	121.2	7.5	87.2	4.1
PFOS	188.8	28.5	145.0	13.2	105.8	2.4
PFNS	108.9	6.3	117.6	2.2	84.1	2.7
PFDS	101.9	4.8	112.1	5.4	87.8	7.3
6∶2 Cl-PFESA	169.0	7.4	122.8	5.0	94.4	5.4
8∶2 Cl-PFESA	106.4	4.9	110.8	5.1	85.7	5.6
10∶2 Cl-PFESA	99.4	5.4	100.3	3.9	115.3	8.6
6∶2 FTS	104.6	2.1	122.8	5.0	119.7	7.3
FBSA	74.9	14.8	76.7	10.1	60.3	3.3
FHxSA	45.9	12.2	78.2	17.9	63.4	2.2
FOSA	56.8	21.4	73.3	3.9	61.2	15.4
*N*-EtFOSAA	94.0	1.2	147.8	10.4	82.6	15.1
*N*-MeFOSAA	91.2	4.1	131.5	8.1	78.9	17.0
GenX	-	-	95.8	22.1	73.7	26.5
ADONA	76.2	3.1	60.0	18.4	69.8	1.6
TMP	98.1	12.0	112.5	8.6	143.3	25.1
TEP	84.3	9.5	117.3	16.2	127.3	11.1
TPrP	79.5	3.2	89.5	13.1	108.7	3.1
TnBP	75.2	3.2	103.2	21.3	110.2	29.0
TEHP	93.9	24.6	105.1	10.6	159.3	4.3
TCEP	108.9	10.6	112.6	5.8	118.8	1.4
TCIPP	116.7	3.4	105.2	15.8	115.4	7.2
TDCPP	90.2	4.2	82.4	14.2	101.2	8.3
V6	126.8	8.5	81.6	14.9	126.4	9.4
TBOEP	86.0	5.1	101.3	16.2	97.8	4.1
TPHP	97.8	11.3	105.1	10.6	119.0	1.3
TMPP	92.3	7.8	54.3	7.3	82.1	8.9
3IPPDPP	87.8	7.8	49.9	13.0	85.7	5.4
CDPP	88.8	4.0	57.9	13.7	106.3	10.7
B3tBPPP	95.6	9.6	77.6	9.7	104.6	5.8
B3IPPP	93.8	9.4	73.9	5.9	107.8	4.9
3tBPDPP	92.0	6.0	48.0	6.7	86.8	9.1
DBP	110.9	18.5	113.9	3.6	127.2	5.2
BEHP	86.5	4.6	121.4	2.9	118.5	7.4
DPHP	102.8	3.9	83.0	11.4	121.6	4.0
BMPP	92.9	13.2	108.2	9.9	119.9	2.7
BBOEP	105.6	15.1	107.3	20.3	110.3	2.8

Low spiked level： 0.2 ng/mL PFAS， 0.5 ng/mL OPEs， di-OPEs； medium spiked level： 2 ng/mL PFAS， 5 ng/mL OPEs， di-OPEs； high spiked level： 4 ng/mL PFAS， 10 ng/mL OPEs， di-OPEs.

### 2.6 实际样品的检测

使用本方法对实际血清样品进行分析，15个人体血清样品中29种PFAS总量为6.71~379 ng/mL，中位含量为22.9 ng/mL。PFHxA、PFHpA、PFOA、PFNA、PFDA、PFUnDA、PFDoDA、PFTrDA、PFBS、PFHxS、PFHpS、PFOS、6∶2 Cl-PFESA、FHxSA及FOSA的检出率高于60.0%。其中PFOA中位含量为8.96 ng/mL，PFOS中位含量为4.07 ng/mL。这一结果与NHANES的统计结果一致^［41］^。17种OPEs总含量为0.015 0~10.5 ng/mL，中位含量为2.81 ng/mL。检出率超过60.0%的化合物是TEP、TnBP和TPHP，其中TnBP中位含量最高，为0.538 ng/mL。相似的，在深圳成年人血清中主要的OPEs也是TnBP^［36］^，不过在济南老年人群血清中检测出的OPEs主要为TCIPP和TPHP^［42］^。由于不同地区环境的影响，人体血清中OPEs的含量存在差异。5种di-OPEs的总含量为<MDL~0.443 ng/mL，中位含量为0.015 0 ng/mL，其中BBOEP的检出率高于50.0%。具体结果见表4。

**表 4:** 人体血清中PFAS、OPEs及di-OPEs的含量

Compound	Content range/ （ng/mL）	Median content/ （ng/mL）	DF/%	Compound	Content range/ （ng/mL）	Median content/ （ng/mL）	DF/%
PFBA	<MDL-0.210	<MDL	13.3	GenX	<MDL	<MDL	0
PFPeA	<MDL	<MDL	0	ADONA	<MDL	<MDL	0
PFHxA	<MDL-0.109	0.0160	60.0	TMP	<MDL	<MDL	0
PFHpA	<MDL-0.172	0.0280	73.3	TEP	<MDL-7.34	0.315	60.0
PFOA	2.28-357	8.96	100.0	TPrP	<MDL	<MDL	0
PFNA	0.365-2.93	0.694	100.0	TnBP	<MDL-5.29	0.538	86.7
PFDA	0.223-1.11	0.356	100.0	TEHP	<MDL	<MDL	0
PFUnDA	0.210-1.76	0.459	100.0	*N*-MeFOSAA	<MDL	<MDL	0
PFDoDA	<MDL-0.147	0.0360	66.7	TCEP	<MDL-0.269	<MDL	40.0
PFTrDA	<MDL-0.468	0.0840	80.0	TCIPP	<MDL-4.04	<MDL	46.7
PFTeDA	<MDL	<MDL	0	TDCPP	<MDL-1.09	<MDL	6.7
PFBS	<MDL-0.0640	0.0120	66.7	V6	<MDL	<MDL	0
PFPeS	<MDL-0.0110	<MDL	33.3	TBOEP	<MDL-1.04	<MDL	33.3
PFHxS	0.155-4.30	1.58	100.0	TPHP	<MDL-0.113	0.0170	66.7
PFHpS	0.0180-0.281	0.0900	100.0	TMPP	<MDL	<MDL	0
PFOS	1.53-9.04	4.07	100.0	3IPPDPP	<MDL	<MDL	0
PFNS	<MDL	<MDL	0	CDPP	<MDL	<MDL	0
PFDS	<MDL	<MDL	0	B3tBPPP	<MDL	<MDL	0
6∶2 Cl-PFESA	0.307-3.50	0.781	100.0	B3IPPPP	<MDL	<MDL	0
8∶2 Cl-PFESA	<MDL-0.0190	<MDL	13.3	3tBPDPP	<MDL	<MDL	0
10∶2 Cl-PFESA	<MDL	<MDL	0	DBP	<MDL-0.150	<MDL	6.7
6∶2 FTS	<MDL	<MDL	0	BEHP	<MDL	<MDL	0
FBSA	<MDL-0.0220	<MDL	20.0	DPHP	<MDL	<MDL	0
FHxSA	<MDL-0.0410	0.0210	66.7	BMPP	<MDL	<MDL	0
FOSA	<MDL-0.0850	0.0220	73.3	BBOEP	<MDL-0.293	0.0150	53.3
*N*-EtFOSAA	<MDL	<MDL	0				
∑PFAS	6.71-379	22.9					
∑OPEs	0.0150-10.5	2.81					
∑di-OPEs	<MDL-0.443	0.0150					

DF： detection frequency.

## 3 结论

本研究建立了通过使用微量血清实现PFAS**、**OPEs和di-OPEs的同时高效萃取方法。操作方法简单，对目标物有良好的回收率和重复性，可以满足对血清中PFAS**、**OPEs及di-OPEs的分析要求。
